# Molecular property prediction by semantic-invariant contrastive learning

**DOI:** 10.1093/bioinformatics/btad462

**Published:** 2023-07-28

**Authors:** Ziqiao Zhang, Ailin Xie, Jihong Guan, Shuigeng Zhou

**Affiliations:** Shanghai Key Lab of Intelligent Information Processing, and School of Computer Science, Fudan University, Shanghai 200438, China; Shanghai Key Lab of Intelligent Information Processing, and School of Computer Science, Fudan University, Shanghai 200438, China; Department of Computer Science and Technology, Tongji University, Shanghai 201804, China; Shanghai Key Lab of Intelligent Information Processing, and School of Computer Science, Fudan University, Shanghai 200438, China

## Abstract

**Motivation:**

Contrastive learning has been widely used as pretext tasks for self-supervised pre-trained molecular representation learning models in AI-aided drug design and discovery. However, existing methods that generate molecular views by noise-adding operations for contrastive learning may face the semantic inconsistency problem, which leads to false positive pairs and consequently poor prediction performance.

**Results:**

To address this problem, in this article, we first propose a semantic-invariant view generation method by properly breaking molecular graphs into fragment pairs. Then, we develop a Fragment-based Semantic-Invariant Contrastive Learning (FraSICL) model based on this view generation method for molecular property prediction. The FraSICL model consists of two branches to generate representations of views for contrastive learning, meanwhile a multi-view fusion and an auxiliary similarity loss are introduced to make better use of the information contained in different fragment-pair views. Extensive experiments on various benchmark datasets show that with the least number of pre-training samples, FraSICL can achieve state-of-the-art performance, compared with major existing counterpart models.

**Availability and implementation:**

The code is publicly available at https://github.com/ZiqiaoZhang/FraSICL.

## 1 Introduction

Nowadays molecular property prediction (MPP) based on deep learning techniques has been a hot research topic of the AI-aided Drug Discovery (AIDD) community (Duvenaud et al. 2015, Gilmer et al. 2017, Jaeger et al. 2018, Xiong et al. 2019, Song et al. 2020, Ying et al. 2021, Zhang et al. 2021). As most of the molecular properties that drug discovery studies concern require *in vivo* or *in vitro* wet-lab experiments to measure, labeled data for MPP tasks are typically scarce, because it is expensive and time-consuming to acquire such data (Altae-Tran et al. 2017). On the contrary, there are large amounts of public available unlabeled data (Gaulton et al. 2017, Irwin et al. 2020, Kim et al. 2021b). Therefore, how to use these large-scale unlabeled molecular data to train deep neural networks to learn better molecular representations for MPP tasks, is of great interest to the AIDD community.

Recently, as self-supervised pre-trained models (PTMs) (e.g. BERT, Devlin et al. 2019; MoCo, He et al. 2020; and SimCLR, Chen et al. 2020) have shown significant superiority in the fields of Natural Language Processing and Computer Vision (CV), self-supervised learning (SSL) has become a mainstream method of utilizing large-scale unlabeled molecular data in MPP study. These SSL methods typically use some inherent features within or between samples to construct pretext tasks, so that unlabeled data can be leveraged to train deep models in a self-supervised learning manner (Jaiswal et al. 2020). Contrastive learning, masked language models and predictive learning are the latest three categories of methods to design pretext tasks in MPP studies (Hu et al. 2020, Rong et al. 2020, Xu et al. 2021, Zhu et al. 2021, 2022, Kim et al. 2021a, Li et al. 2022, Wang et al. 2022), which have pushed the boundaries of predictive accuracy on several benchmarks. Among the three categories, contrastive learning methods aim at learning representations through contrasting positive data pairs against negative ones (Wang et al. 2022). Original molecular structures are augmented into multiple views, and views generated from the same molecule are typically used as positive data pairs, while views of different molecules are taken as negative ones (Wang et al. 2022).

The way to generate molecular views is crucial to the design of contrastive learning pretext tasks for molecular representation learning. As a kind of special objects, molecules can be represented by different methods, including molecular fingerprints (Rogers and Hahn 2010), SMILES (Weininger 1988), IUPAC (Favre and Powell 2013), and molecular graphs. These different molecular representations therefore can naturally be leveraged as views for contrastive learning. The DMP (Zhu et al. 2021) and MEMO (Zhu et al. 2022) models are designed in this way. Following practices in CV, another widely used category of methods tries to *add noise* into molecular structures to generate transformations as views of the original molecules. These noise-adding operations include deleting atoms, replacing atoms, deleting bonds, deleting subgraph structures etc. MolCLR (Wang et al. 2022) and GraphLoG (Xu et al. 2021) are such representative models.

**Figure 1.**
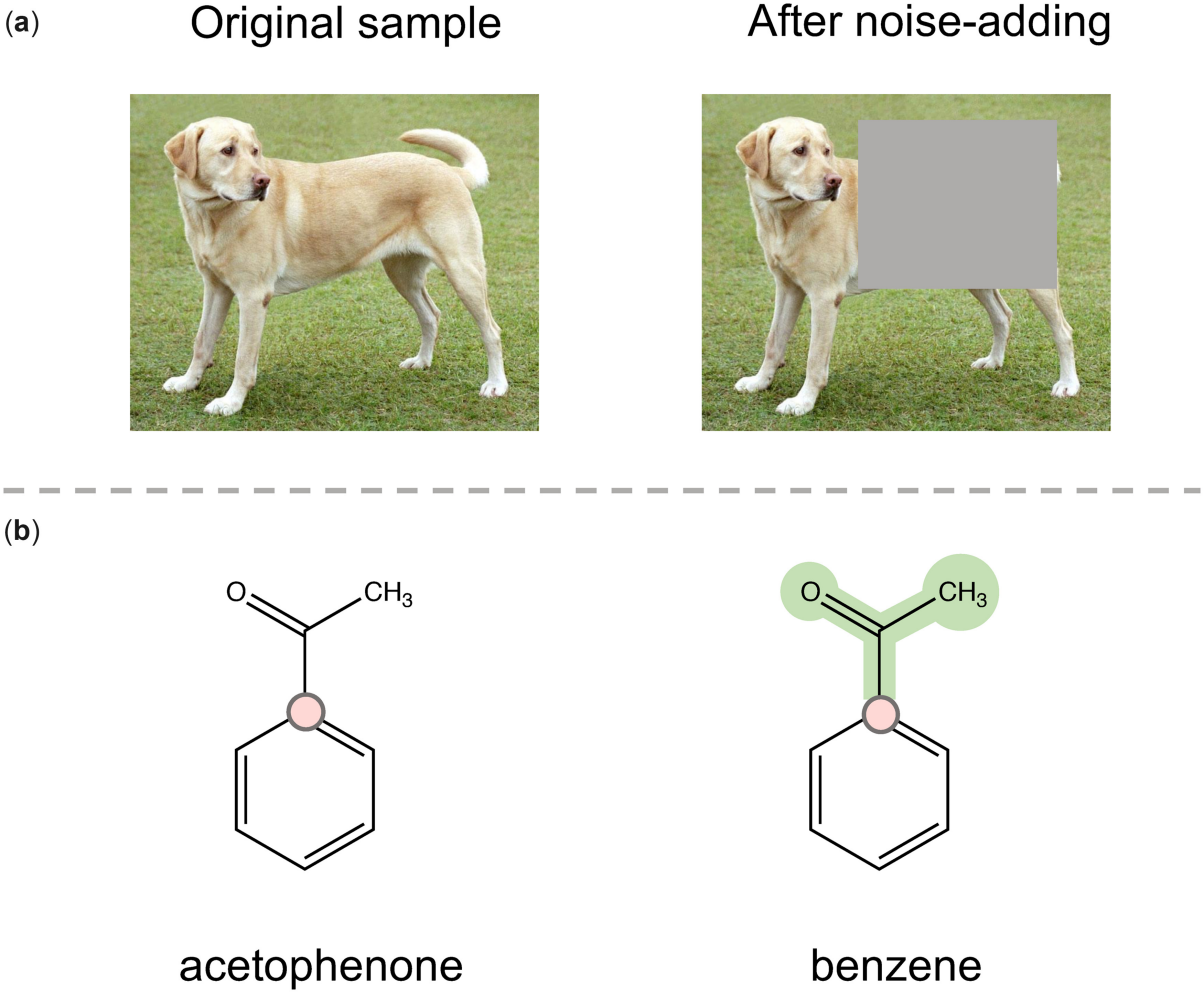
Illustration of the influence of noise-adding operation to the semantic of generated view. (a) After adding noise to the image by randomly masking some area, semantic of the image does not change, i.e. it still represents a dog. (b) By adding noise into the molecular graph of acetophenone via masking a subgraph, the generated graph represents a completely different molecule *benzene* with different molecular properties. The chemical semantic is completely changed.

Although the effectiveness of noise-adding methods for view generation have been proved in CV studies (Chen et al. 2020, Grill et al. 2020), when applying these methods into MPP tasks, a fact that has not been noticed by the researchers is that molecules are very sensitive to noise. Arbitrarily modifying the topological structure of a molecule with noise may lead to producing a structure that represents a totally different molecule. For instance, as shown in Fig. 1a, adding noise into an image by randomly masking some area will not change the semantic of the generated view, which is still a yellow dog. However, in Fig. 1b, deleting a subgraph of an acetophenone’s molecular graph may lead to a benzene, indicating that the chemical semantic of the graph is completely changed. Furthermore, small differences between molecular structures may lead to dramatic changes in the properties of modified molecules, including both bio-activity and other physio-chemical properties. Concretely, from the PubChem database (Kim et al. 2021b) we can find that the LogP value of acetophenone in Fig. 1b is 1.58, while that of benzene is 2.13. The difference is almost 35%. Therefore, it is unreasonable to treat these two views (two molecules exactly) as a positive pair for contrastive learning.

Aiming at solving this semantic inconsistency problem, this paper proposes a **Fra**gment-based **S**emantic-**I**nvariant **C**ontrastive **L**earning molecular representation model, named FraSICL. A semantic-invariant molecular view generation method is developed, in which a molecular graph is properly broken into fragments to change the graph topology fed into the backbone Graph Neural Network (GNN) encoder, while preserving the molecular structural information. A multi-view fusion mechanism is introduced to FraSICL to make better use of the information contained in different views. In addition, an auxiliary similarity loss is designed.

Our contribution are summarized as follows:

- We raise the semantic inconsistency problem in molecular view construction for molecular constrastive learning and develop an effective method to generate semantic-invariant graph views by changing the graph topology while preserving the structural information of molecules.
- We propose a novel Fragment-based Semantic-Invariant Contrastive Learning molecular representation model for effective MPP, which is also equipped with a multi-view fusion mechanism and an auxiliary similarity loss to better leverage the information contained in unlabeled pre-training data.
- Extensive experiments show that compared with SOTA pre-trained MPP models, the proposed FraSICL can achieve better prediction accuracy on downstream target tasks with less amounts of unlabeled pre-training data.

## 2 Materials and methods

In this section, we first formally define semantic-invariant molecular view, then propose a semantic-invariant molecular view generation method and a multi-view fusion scheme. Finally, the structure of the Fragment-based Semantic-Invariant Contrastive Learning (FraSICL) molecular representation model and its loss functions are introduced.

### 2.1 Semantic-invariant molecular view

In the Section 1, we have given an example to illustrate how noise-adding operations may lead to semantic inconsistency and consequently false positive pairs. Here, we formally define *semantic-invariant molecular view*.

Given a molecule *m* and its molecular graph *G* = $\left\{V,E,X_{\mathrm{atom}},X_{\mathrm{bond}}\right\}$ (hydrogen-depleted) where *V* denotes the set of nodes that represent the atoms, *E* denotes the set of edges between nodes, representing the bonds. $X_{\mathrm{atom}}$ and $X_{\mathrm{bond}}$ are feature matrix of atoms and bonds respectively. A *transformation function* F(⋅) is used to generate a *molecular graph view* (or simply *molecular view*) $G^{\prime }$ of *G*, i.e. $G^{\prime }$ = F(G) and $G^{\prime }$=$\left\{V^{\prime },E^{\prime },X_{\mathrm{atom}}^{\prime },X_{\mathrm{bond}}^{\prime }\right\}$. In what follows, we first define two types of semantic inconsistent views.

Definition 1 (Semantic-conflict view) If there is another molecule $m_{2}$ whose molecular graph is $G_{2}$, and $G_{2}$=$G^{\prime }$=F(G), i.e. the view $G^{\prime }$ of *m* is the same as the molecular graph $G_{2}$ of $m_{2}$, then we say $G^{\prime }$ is a *semantic-conflict view* of *m* with regard to (w.r.t.) $m_{2}$.

Definition 2 (Semantic-ambiguity view) If there exists another molecule $m_{2}$ whose molecular graph is $G_{2}$, and $G_{2}^{\prime }$=$F(G_{2})$=$G^{\prime }$=F(G), i.e. the view $G^{\prime }$ of *m* is the same as a view $G_{2}^{\prime }$ of $m_{2}$. Then, we say $G^{\prime }$ is a *semantic-ambiguity view* of molecule *m* w.r.t. molecule $m_{2}$.

Both semantic-conflict views and semantic-ambiguity views will lead to false positive pairs for molecular representation contrastive learning. For example, assume that a GNN g(⋅) serves as an encoder to embed the molecular graphs into latent graph embeddings $h_{G}=g(G)$. If the graph encoder is deterministic, i.e. similar input graphs will lead to similar graph embeddings, it is obvious that, for molecule *m*, if it has a semantic-conflict view $G^{\prime }$ w.r.t. molecule $m_{2}$, i.e., $G^{\prime }$=F(G)=$G_{2}$, then the representation of $G^{\prime }$ embedded by the GNN will be the same as that of $G_{2}$. That is, $h_{G^{\prime }}$=$g(G^{\prime })$=$g(G_{2})$=$h_{G_{2}}$. In this case, as $h_{G}$ and $h_{G^{\prime }}$ are considered as a positive pair in contrastive learning, $h_{G}$ and $h_{G_{2}}$ are consequently used as a positive pair. In another word, the contrastive loss will implicitly make the representations of molecule *m* and $m_{2}$ to be close. However, as claimed before, the molecular properties of different molecules may be greatly different, so that they cannot be used as a positive pair for contrastive learning. Therefore, semantic-conflict views will lead to false positive pairs and degrade learning performance.

On the other hand, if a semantic-ambiguity view is generated as defined in Definition 2, i.e. $F(G_{2})$=$G^{\prime }$=F(G), the contrastive loss will make $h_{G}$ and $h_{G^{\prime }}$, $h_{G_{2}}$ and $h_{G^{\prime }}$ to be close in the embedding space, thus $h_{G}$ and $h_{G_{2}}$ to be close, too. So semantic-ambiguity views will also lead to false positive pairs.

To boost the performance of contrastive learning for MPP, we should avoid the generation of both semantic-conflict views and semantic-ambiguity views. That is, we generate only semantic-invariant views, which are defined as follows:

Definition 3 (Semantic-invariant view) Given a view $G^{\prime }$ of molecule *m* with graph *G*, if $G^{\prime }$ is neither a semantic-conflict view nor a semantic-ambiguity view w.r.t. any other molecules, then we say $G^{\prime }$ is a *semantic-invariant view* of *m*.

### 2.2 Semantic-invariant view generation

According to Definition 3, semantic-invariant views should be neither semantic-conflict views nor semantic-ambiguity views. Besides, they should also be discriminative so that they can be encoded into different representations by neural network encoders. To achieve these goals, we propose a semantic-invariant view generation method.

Although adding noise to molecules may lead to semantic inconsistency problems, it is still an efficient way to generate discriminative views for molecular graphs. Therefore, strategies to add noise should be carefully designed. Recently, researchers have discovered that graph rewiring, i.e. adding or removing bonds to or from the input graph to make the information propagating along a different topology inside a GNN encoder while preserving the original structural information by encodings (Ying et al. 2021), will overcome some shortcomings of GNNs (Alon and Yahav 2021, Brüel-Gabrielsson et al. 2022, Topping et al. 2022). Inspired by these findings, our semantic-invariant view generation method is designed as follows:

Given a molecule *m*, its molecular graph can be denoted as an annotated graph $G=\{V,E,X_{\mathrm{atom}},X_{\mathrm{bond}}\}$. The atom feature matrix $X_{\mathrm{atom}}$ and the bond feature matrix $X_{\mathrm{bond}}$ are computed according to Supplementary Table S1. Then, randomly remove one of the acyclic single bonds $e_{ij}$ from *E*, we obtain $G^{\prime }=\{V,E^{\prime },X_{\mathrm{atom}},X_{\mathrm{bond}}\}$ where $E^{\prime }$ = $E-\{e_{ij}\}$. We accept $G^{\prime }$ as a view to be generated, i.e. a semantic-invariant view. As the graph $G^{\prime }$ consists of two disconnected molecular graph fragments, it is also called *fragment-pair view*.

Though this method seems similar to some other edge removal methods, this carefully designed strategy will bring advantages from the following perspectives:

1. By randomly removing one edge from edge set *E*, $G^{\prime }$ is a different graph (disconnected) from the original molecular graph *G*. So the GNN encoder will generate a different embedding for $G^{\prime }$, i.e. $h_{G^{\prime }}\neq h_{G}$. Thus, the generated view for a molecule is discriminative.
2. Since only the edge set *E* is modified in $G^{\prime }$, the topology described by $\left\{V,E^{\prime }\right\}$ is not consistent with the information encoded in $X_{\mathrm{atom}}$. Specifically, degrees of node *i* and *j* in graph $G^{\prime }$ are lower than the numbers of bonds of *i* and *j* recorded in $X_{\mathrm{atom}}$. In another word, our method makes sure that $G^{\prime }$ is **NOT** a valid molecular graph of any molecule. Therefore, $G^{\prime }$ cannot be a semantic-conflict view w.r.t. any molecule according to Definition 1.
3. Since only **ONE** single bond is removed in view $G^{\prime }$, this discrepancy between $E^{\prime }$ and $X_{\mathrm{atom}}$ can only be discovered at nodes *i* and *j*, and the deviation is only 1. So, the removed edge can only be a single bond between *i* and *j*. Thus, there is no other molecular graph $G_{2}$ that can generate the same $G^{\prime }$ by this method. Therefore, $G^{\prime }$ cannot be a semantic-ambiguity view w.r.t. any molecule according to Definition 2. In summary, $G^{\prime }$ is a semantic-invariant view.
4. Compared with randomly breaking one arbitrary bond of a molecular graph, our method can generate graph fragments that may correspond to some chemical meaningful functional groups, since acyclic single bonds can serve as boundaries between functional groups (Zhang et al. 2021). And experimental results in our previous work have shown that learning representations with such graph fragments can achieve good predictive performance in MPP tasks (Zhang et al. 2021).

### 2.3 Model structure

**Figure 2.**
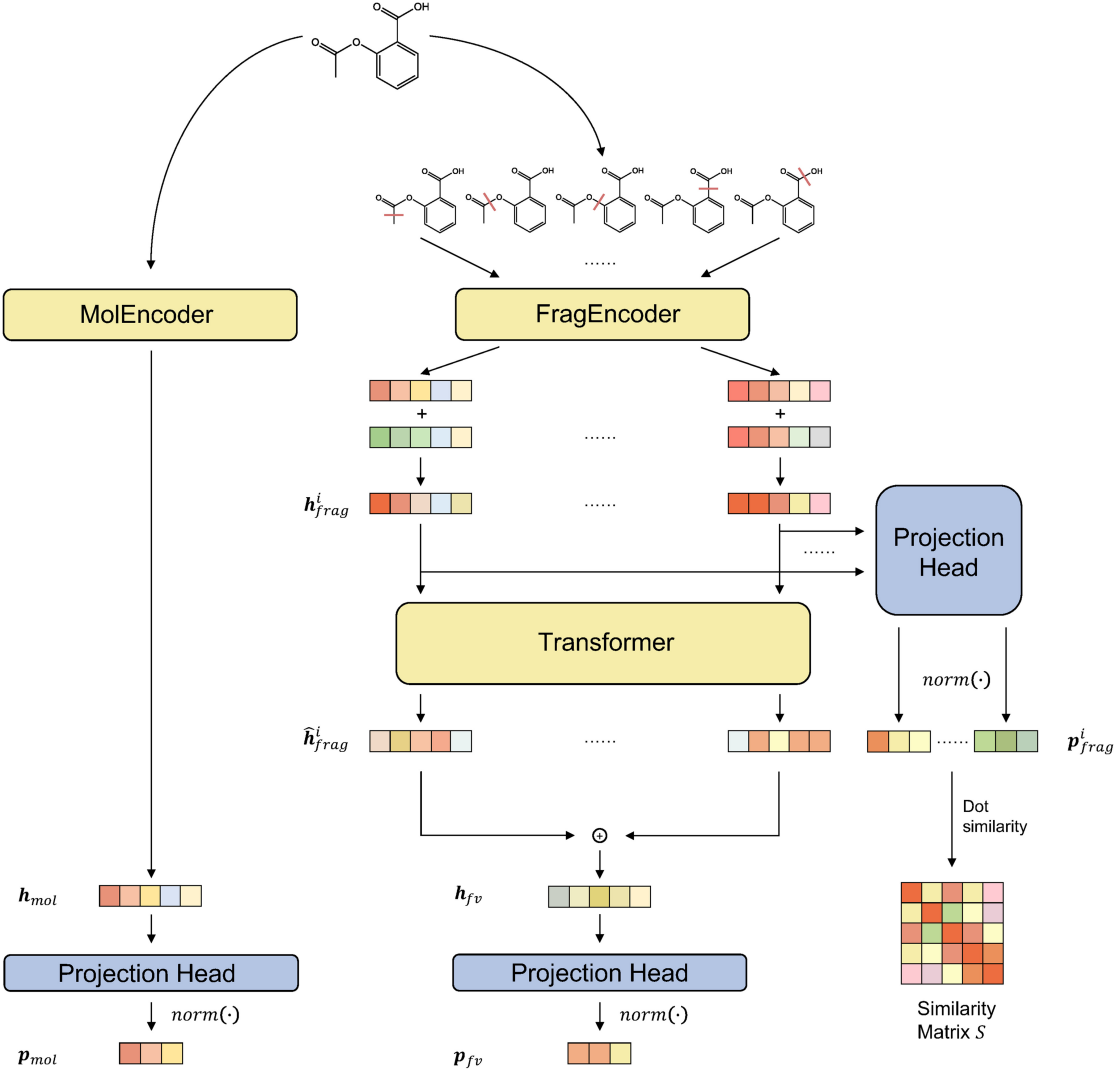
Structure of the FraSICL model.

With the proposed semantic-invariant view generation method, the structure of the FraSICL model is shown in Fig. 2. Given a molecule *m* with molecular graph $G_{\mathrm{mol}}=\{V,E,X_{\mathrm{atom}},X_{\mathrm{bond}}\}$, the model computes the representations of two views via two branches: the left branch is the *molecule view branch* for generating molecular view, and the right one is the *fragment view branch* for generating the fragment view.

In the molecule view branch, a GNN $g_{\mathrm{mol}}(\cdot )$ is used as an encoder, denoted by **MolEncoder**, to capture the representation of the molecular graph $h_{\mathrm{mol}}=g_{\mathrm{mol}}(G_{\mathrm{mol}})$. Attentive FP (Xiong et al. 2019) is employed as the graph encoder in this work. Then, $h_{\mathrm{mol}}$ is fed into a projection head (Wang et al. 2022) $l_{\mathrm{mol}}(\cdot )$ and a regularization function norm(⋅) to produce the projection of the molecule view $p_{\mathrm{mol}}=\mathrm{norm}(l_{\mathrm{mol}}(h_{\mathrm{mol}}))$. Structure of a projection head is shown in Supplementary Fig. S1. And the regularization function is $\mathrm{norm}(v)=\frac{v}{\left||v|\right|}$.

For the fragment view branch on the right, instead of randomly generating views as other contrastive learning models do, in FraSICL we enumerate and break all of the $N_{b}$ breakable acyclic single bonds by the proposed method to generate $N_{b}$ fragment-pair views $G_{\mathrm{frag}}^{i}$=$\left\{V,E_{i},X_{\mathrm{atom}},X_{\mathrm{bond}}\},i\in \{1,\ldots ,N_{b}\right\}$. Another GNN $g_{\mathrm{frag}}(\cdot )$ is used as an encoder, denoted by **FragEncoder**, to compute the representation of each fragment-pair view $h_{\mathrm{frag}}^{i}=g_{\mathrm{frag}}(G_{\mathrm{frag}}^{i})$. Attentive FP is also used here. Note that since there are two disconnected components in each fragment-pair view $G_{\mathrm{frag}}^{i}$, $g_{\mathrm{frag}}(\cdot )$ will read out these two subgraphs separately and produce two subgraph embeddings. The representation of a fragment-pair view is obtained by element-wisely adding two corresponding subgraph embeddings.

Then, to better leverage all of the information related to functional groups contained in the $N_{b}$ fragment-pair views, a multi-view fusion mechanism is introduced. Specifically, a Transformer encoder T(⋅) is employed, which uses the representations of fragment-pair views $h_{\mathrm{frag}}^{i}$ as input tokens, and computes the interaction relationships between the fragment-pair views by the multi-head attention mechanism. The resulting attention scores serve as weights to fuse the representations and obtain ${\hat{h}}_{\mathrm{frag}}^{i}$. Then, we can get the representation of *fragment view* $h_{fv}=\sum_{i=1}^{N_{b}}{\hat{h}}_{\mathrm{frag}}^{i}$. Finally, the representation $h_{fv}$ of fragment view goes through a projection head $l_{fv}(\cdot )$ and a normalization layer to get $p_{fv}=\mathrm{norm}(l_{fv}(h_{fv}))$. These two projections $p_{\mathrm{mol}}$ and $p_{fv}$ are used to calculate contrastive loss. And when finetuning on downstream tasks, the model will output either of the representations $h_{\mathrm{mol}}$ and $h_{fv}$ of a molecule to serve as learned molecular representation. A downstream prediction head f(⋅) will use this representation as input, and predict the molecular property by $y=f(h_{\mathrm{mol}})$ or $y=f(h_{fv})$.

In addition, representations $h_{\mathrm{frag}}^{i}$ of $N_{b}$ fragment-pair views of a molecule goes through another projection head and a normalization layer to produce projection $p_{\mathrm{frag}}^{i}=\mathrm{norm}(l_{\mathrm{frag}}(h_{\mathrm{frag}}^{i}))$. Inner product of these projections are computed to generate a similarity matrix $S=\{s_{ij}|s_{ij}=<p_{\mathrm{frag}}^{i},p_{\mathrm{frag}}^{j}>\},S\in R^{N_{b}\times N_{b}}$, where <⋅,⋅> denotes the inner product of two vectors. This similarity matrix *S* will be used to compute auxiliary similarity loss, which will be introduced in the next sub-section.

### 2.4 Loss functions

**Figure 3.**
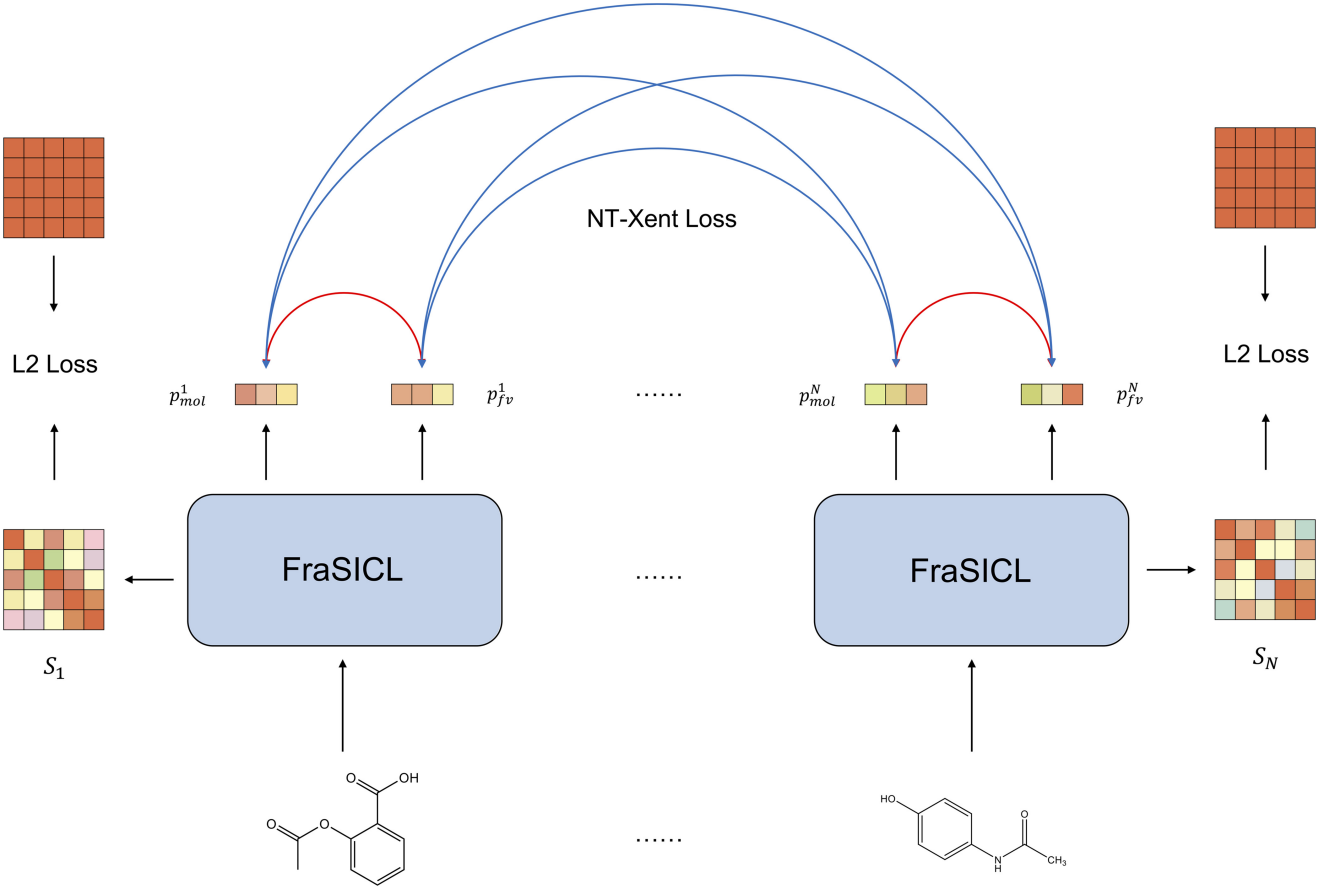
Illustration of FraSICL training. FraSICL is trained by both NT-Xent contrastive loss and an auxiliary similarity loss. In the contrastive loss, two projections of a molecule are treated as a positive pair, which is highlighted by red lines in the figure. Projections of other molecules in a batch are considered as negative pairs, which is shown by blue lines. For each molecule, L2 loss is computed between the similarity *S* and an all-one matrix as auxiliary similarity loss.

**NT-Xent loss.** The training of FraSICL in the pre-training phase is illustrated in Fig. 3. Here, given a batch of *N* molecules, the model will calculate projections $p_{\mathrm{mol}}$ and $p_{fv}$ of each molecule. Then, contrastive learning is performed between all samples in a batch. The view pair (i.e. molecule view and fragment view) of each sample is a positive pair, as shown by the red lines, and the view pairs of different samples in the batch are negative pairs, as shown by the blue lines in Fig. 3. The NT-Xent Loss is used for contrastive learning: where inner product similarity is adopted for $\mathrm{sim}(p_{\mathrm{mol}}^{i},p_{fv}^{i})$, 1{k≠i} is an indicator function with value 1 iff k≠i, and τ is a temperature parameter. The sum of all contrastive losses of a batch of molecules is denoted as $L_{\mathrm{clr}}=\sum_{i=1}^{N}\left(L_{\mathrm{mol}}^{i}+L_{fv}^{i}\right)$.


(1) $$L_{\mathrm{mol}}^{i}=\text{log}\;\frac{e^{\left(\frac{\mathrm{sim}(p_{\mathrm{mol}}^{i},p_{fv}^{i})}{\tau }\right)}}{\sum_{k=1}^{N}1\{k\neq i\}(e^{\left(\frac{\mathrm{sim}(p_{\mathrm{mol}}^{i},p_{\mathrm{mol}}^{k})}{\tau }\right)}+e^{\left(\frac{\mathrm{sim}(p_{\mathrm{mol}}^{i},p_{fv}^{k})}{\tau }\right)})},$$

(2) $$L_{fv}^{i}=\text{log}\;\frac{e^{\left(\frac{\mathrm{sim}(p_{\mathrm{mol}}^{i},p_{fv}^{i})}{\tau }\right)}}{\sum_{k=1}^{N}1\{k\neq i\}(e^{\left(\frac{\mathrm{sim}(p_{fv}^{i},p_{\mathrm{mol}}^{k})}{\tau }\right)}+e^{\left(\frac{\mathrm{sim}(p_{fv}^{i},p_{fv}^{k})}{\tau }\right)})},$$

**Similarity loss.** In addition, although the representations of different fragment-pair views have been fused, from the perspective of contrastive learning, the representations of different fragment-pair views of the same molecule should also be as close as possible. And as demonstrated in our previous study (Zhang et al. 2021), representations of some fragment pairs of a molecule are highly predictive on the downstream tasks, while some others are less effective. So, we hope that the representations of fragment-pair views can exploit information from each other to train the GNN encoder to extract better representations. To this end, an additional auxiliary loss $L_{\mathrm{sim}}$ is introduced to improve the similarity between representations of fragment-pair views of a molecule, based on the similarity matrix *S*. Since the inner product of two normalized vectors is equivalent to cosine similarity, and the maximum value of cosine similarity is 1. Assume that a molecule *k* has $N_{b}^{k}$ fragment-view pairs, and the elements of the similarity matrix are $s_{ij}^{k}=<p_{\mathrm{frag}}^{k,i},p_{\mathrm{frag}}^{k,j}>$, our auxiliary similarity loss of molecule *k* is: i.e. as shown in Fig. 3, the sum of L2 loss between each element of the similarity matrix *S* and that of an all-one matrix. Denote the similarity loss of a batch of molecules as $L_{\mathrm{sim}}=\sum_{k=1}^{N}L_{\mathrm{sim}}^{k}$.


(3) $$L_{\mathrm{sim}}^{k}=\frac{1}{{\left(N_{b}^{k}\right)}^{2}}\sum_{i=1}^{N_{b}^{k}}\sum_{j=1}^{N_{b}^{k}}{\left(s_{ij}^{k}-1\right)}^{2},$$

**Total loss.** The total loss for pre-training the FraSICL model is: where γ is a hyper-parameter to adjust the influence of the auxiliary similarity loss.


(4) $$L=\gamma L_{\mathrm{sim}}+L_{\mathrm{clr}},$$

## 3 Experimental results and analysis

### 3.1 Baseline experiments

**Experimental setting.** To construct the pre-training dataset, 200K molecules are randomly sampled from the pre-training dataset of MolCLR, where 10 million molecules are gathered from the PubChem database (Kim et al. 2021b). The amount of pre-training data is generally smaller than that of the other baseline models, as shown in Supplementary Table S2. Five percent of the pre-training data are randomly selected as the validation set for model selection. Seven downstream tasks from MoleculeNet (Wu et al. 2018) are used as downstream target tasks for the baseline experiments. Scaffold splitting is used on each downstream task, with an 8:1:1 ratio for the training/validation/test sets.

When transferring a pre-trained FraSICL model to the target tasks, different strategies can be applied, including which branch of the model is used for producing molecular representations, and whether to finetune the PTM on target tasks. In the baseline experiments, we adopt the more complex fragment view branch for molecular representations, and finetune the model together with prediction head on the target tasks.

**Compared baseline models.** Seven state-of-the-art self-supervised pre-training models for molecular representation learning are used as baseline models for comparison, including MolCLR (Wang et al. 2022), DMP (Zhu et al. 2021), MEMO (Zhu et al. 2022), GROVER (Rong et al. 2020), GraphLoG (Xu et al. 2021), PretrainGNNs (Hu et al. 2020), and KPGT (Li et al. 2022). Detailed introduction of these baseline models are presented in the Supplementary Material. The experimental results are given in Table 1, where the data of baseline models are cited from the original papers of these models. The best score on each dataset is bold, and the second-best is underlined.

**Table 1.** Results of performance comparison between FraSICL and major existing models on 7 downstream MPP tasks.

Model	BACE classification	BBBP classification	ClinTox classification	Tox21 classification	ESOL regression	FreeSolv regression	Lipop regression
MolCLR	0.890 ± 0.003	0.736 ± 0.005	0.932 ± 0.017	0.798 ± 0.007	1.110 ± 0.010	2.200 ± 0.200	0.650 ± 0.080
DMP-TF	0.893 ± 0.009	0.781 ± 0.005	0.950 ± 0.005	0.788 ± 0.005	0.700 ± 0.084		
MEMO	0.826 ± 0.003	0.716 ± 0.010	0.816 ± 0.037	0.767 ± 0.004	0.984 ± 0.034		0.707 ± 0.001
GROVER	0.894 ± 0.028	0.940 ± 0.019	0.944 ± 0.021	0.831 ± 0.025	0.831 ± 0.120	1.544 ± 0.397	**0.560** ± **0.035**
PretrainGNNs	0.845 ± 0.007	0.687 ± 0.013	0.726 ± 0.015	0.781 ± 0.006			
GraphLoG	0.835 ± 0.012	0.725 ± 0.008	0.767 ± 0.033	0.757 ± 0.005			
KPGT	0.855 ± 0.011	0.908 ± 0.010	0.946 ± 0.022	**0.848** ± **0.013**	0.803 ± 0.008	2.121 ± 0.837	0.600 ± 0.010
FraSICL	**0.896** ± **0.010**	**0.948** ± **0.003**	**0.957** ± **0.011**	0.807 ± 0.006	**0.626** ± **0.008**	**1.094** ± **0.027**	0.581 ± 0.013

The best score on each dataset is bold, and the second-best is underlined.

**Results and analysis.** As shown in Table 1, FraSICL achieves the best predictive performance on five of the seven downstream MPP tasks, and the second on another one. As the number of pre-training samples used by FraSICL is only 200K, which is the least among these compared baseline models, the results show that FraSICL can make better use of the information contained in the graph fragments of molecules to produce molecular representations with better predictive performance. Compared with the MEMO model that uses the same amount of pre-training data, the predictive performance of FraSICL on the seven downstream tasks is significantly improved, even exceeds 20 percentage on the BBBP dataset. And compared with the models such as GROVER and DMP-TF, FraSICL can achieve comparable or even higher predictive performance with only about 1/50 training samples. These results show the superiority of FraSICL over the existing models on MPP tasks.

### 3.2 Experiments under different transferring settings

**Experimental setting.** In the baseline experiments, we choose finetuning the more complex and predictive fragment view branch as the transferring setting. In this section, other transferring settings are tested, i.e. the combinations of different branches and different fine-tuning strategies. Experiments are carried out on the BBBP, ClinTox, ESOL, and FreeSolv datasets. Four transferring settings are evaluated, which are denoted as FraSICL-ft-mol, FraSICL-ft-frag, FraSICL-fr-mol, FraSICL-fr-frag, where *ft* represents finetuning the PTM, *fr* represents freezing the PTM, *mol* indicates using molecule views and *frag* indicates using fragment views.

**Results and analysis.** The experimental results are presented in Table 2. Since the two branches of FraSICL are asymmetric, the structure of the fragment view branch is more complex and has stronger learning capability. Thus, as is revealed by the experimental results, FraSICL-ft-frag achieves the best performance on three of the four target tasks. However, a more complex model structure indicates that it is more likely to suffer from overfitting on the downstream tasks. So, when transferring to the FreeSolv dataset with only 642 samples, the performance of FraSICL-ft-frag is slightly inferior to that of FraSICL-ft-mol. In addition, compared with freezing the PTM, the finetuning model allows the PTM to obtain information about specific molecular properties from the supervised loss, thereby the generated molecular representations are more relevant to the target task. Thus, a larger improvement on the performance is achieved.

**Table 2.** Predictive performance of four FraSICL model variants with different transferring settings on four MPP tasks.

Model	BBBP classification	ClinTox classification	ESOL regression	FreeSolv regression
FraSICL-fr-mol	0.852 ± 0.003	0.691 ± 0.007	1.321 ± 0.002	2.548 ± 0.006
FraSICL-fr-frag	0.803 ± 0.010	0.628 ± 0.020	1.594 ± 0.217	2.293 ± 0.009
FraSICL-ft-mol	0.917 ± 0.006	0.906 ± 0.023	0.758 ± 0.029	**1.085** ± **0.059**
FraSICL-ft-frag	**0.948** ± **0.003**	**0.957** ± **0.011**	**0.626** ± **0.008**	1.094 ± 0.027

The best score on each dataset is bold.

### 3.3 Influence of the auxiliary similarity loss

**Motivation.** The auxiliary similarity loss, i.e. Equation (3), is designed for making the fragment-pair views to learn from each other to better leverage the information encoded in different fragment-pair views. However, it is intuitive that when the auxiliary loss takes an excessively dominant role in the total training loss, the model may tend to generate exactly the same representation vectors for different fragment-pair views to decrease the similarity loss. On this occasion, the representation vectors of fragment-pair views will not contain any information about the topological structure, showing a model collapse phenomenon. Thus, the influence of the hyperparameter γ is crucial.

**Experimental setting.** To test the influence of the auxiliary similarity loss, γ is set to 0.1, 0.01, 0.005, and 0, respectively, where γ=0 indicates training without the similarity loss. This experiment can be regarded as an ablation study on γ. Here, the transferring setting is the same as the baseline experiments, i.e. finetuning the fragment view branch.

**Results and analysis.** Results are presented in Table 3. As can be seen from Table 3, the auxiliary similarity loss has an obvious impact on the predictive performance. When γ=0, i.e. training without the similarity loss, the predictive performance is not superior to that of the other three models trained with the auxiliary similarity loss, which demonstrates that the auxiliary similarity loss can indeed promote the model to produce more predictive representations. And when γ=0.1, the model achieves even worse results than γ=0 on three of the four downstream tasks, which reveals that a large value of γ may make the similarity loss be harmful to the model and lead to performance degradation.

**Table 3.** Results on the influence of hyperparameter γ.

γ	BBBP classification	ClinTox classification	ESOL regression	FreeSolv regression
0.1	0.890 ± 0.023	0.916 ± 0.008	0.734 ± 0.028	1.151 ± 0.098
0.01	**0.948** ± **0.003**	**0.957** ± **0.011**	**0.626** ± **0.008**	**1.094** ± **0.027**
0.005	0.927 ± 0.008	0.912 ± 0.012	0.667 ± 0.024	1.148 ± 0.015
0	0.906 ± 0.009	0.880 ± 0.030	0.662 ± 0.051	1.138 ± 0.092

## 4 Conclusion

This article focuses on the semantic inconsistency problem that may occur when using noise-adding operations to generate new views for contrastive learning in self-supervised MPP studies. To solve this problem, this paper first defines semantic-invariant molecular view by introducing two types of semantic inconsistent views that may lead to false positive pairs and consequently poor performance. Then, a semantic-invariant view generation method is proposed. The views generated by this method will not cause semantic inconsistency, and even may correspond to some chemical meaningful functional groups. Thus, this method is expected to help generating more predictive molecular representations.

Based on the semantic-invariant views, a Fragment-based Semantic-Invariant Contrastive Learning (FraSICL) molecular representation model is developed. FraSICL is an asymmetric model with two branches, the molecule view branch and the fragment view branch. A multi-view fusion mechanism is also introduced to make better use of the information contained in the views of different fragment pairs. Furthermore, an auxiliary similarity loss is designed to train the model to produce better representations.

Baseline experiments are conducted on seven target tasks, and experimental results show that FraSICL achieves state-of-the-art predictive performance with the least amount of pre-training data. Further experiments demonstrate that model finetuning is effective in boosting performance and the auxiliary similarity loss can improve the predictive accuracy if a proper hyperparameter γ is selected. These findings reveal that FraSICL can make better use of the information of pre-training samples and generate representations with superior predictive performance.

## Supplementary Material

btad462_Supplementary_Data
