# Supplementary material for: Molecular property prediction by semantic-invariant contrastive learning
Source: Bioinformatics. 2023 Jul 28;39(8):btad462. doi: 10.1093/bioinformatics/btad462 (PMC10397537; doi:10.1093/bioinformatics/btad462)
Supplement: btad462_Supplementary_Data [file btad462_supplementary_data.pdf]

Supplementary materials

S1 Structure of Projection Heads

The structure of a projection head is shown in Fig. S1.

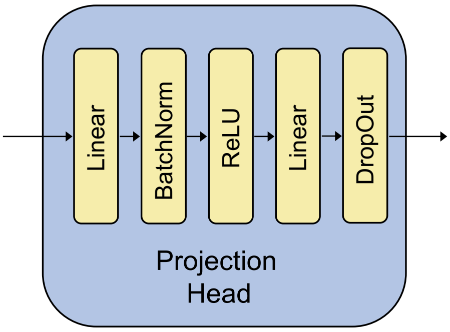

Figure S1: The structure of a projection head. A projection head consists of a stack of linear layer, BN layer, activation layer, linear layer and dropout layer.

S2 Properties of Atoms and Bonds.

Table S1: Properties of atoms and bonds in X_{atom} and X_{bond}.

Indices of atomic features	Description
0-15	Atomic symbol, a one-hot vector of [B,C,N,O,F,Si,P,S,Cl,As,Se,Br,Te,I,At,metal]
16-21	Number of bonds
22	Electrical charge
23	Number of radical electrons
24-29	Hybridization, a one-hot vector of [sp, sp^2, sp^3, sp^3d, sp^3d^2, other]
30	Aromaticity
31-35	Number of connected hydrogens
36	Whether the atom is a chiral center
37-38	Chirality type, a one-hot vector of [R,S]
**Indices of bond features**	**Description**
0-3	Bond type, a one-hot vector of [single, double, triple, aromatic]
4	Whether the bond is conjugated
5	Whether the bond is in a ring
6-9	Stereo, a one-hot vector of [StereoNone, StereoAny, StereoZ, StereoE]

S3 Introduction of baseline pre-trained models

In this work, 7 pre-trained models are involved as baseline models for comparison. In this section, we will give a brief introduction for each of these baseline models.

Inspired by the practice of contrastive learning in the CV realm, MolCLR [4] is proposed to promote the exploitation of unlabeled molecule datasets. In this framework, each molecule is augmented into two views by *adding-noise*, and the encoder model is trained to contrast these views by NT-Xent Loss. Three methods are proposed to generate views, including atom masking, bond deletion and subgraph removal. As discussed in the Introduction section, these noise-adding methods will encounter molecular semantic inconsistency problem.

Different molecular representations can be naturally used as molecular views for contrastive learning. In DMP [6], SMILES and molecular graph are used as two views of a molecule for contrastive learning. A Transformer and a GNN are used as backbone encoders to learn representations for these two views, respectively. MEMO [7] proposes to use four molecular representations as views, including 2D topology graph, 3D geometry graph, Morgan fingerprints and SMILES strings. An attention network is involved to weighted average embeddings of the four views, and contrastive learning is performed by aligning this aggregated embedding with all view-specific embeddings.

In the paper of GROVER [3], two pretext tasks are proposed to train a Transformer-based backbone model for representation learning. For one task, models are trained to predict a randomly masked neighborhood based on the embedding of its central node. For the other one, models are trained to predict the motifs of a given molecular graph, which is calculated by the RDKit package. Therefore, these two pretext tasks can be classified into masked language models category and predictive learning category. In addition, two transformer models equipped with dynamic message passing network, where variational number of layers provide a dynamic receptive field, are exploited to encode the node and edge embedding, respectively.

In GraphLoG framework [5], graph embedding in the latent space are refined from both local and global structure aspects. Specifically, contrastive learning is conducted on the local structure perspective. Models are trained to make structural similar samples closer in the latent space, and enlarge the distance between negative pairs. Views $G' = (V', E', X_{V'}, X_{E'})$ are generated by randomly masking a part of node/edge attributes of the molecular graph. Thus, although the authors have claimed that the GraphLoG is expected to construct a locally smooth latent space by aligning the embedding of correlation graphs/subgraphs, it will encounter the semantic inconsistency problem. And from the global structure perspective, graph embedding is modeled by hierarchical prototypes with maximum likelihood estimation by EM algorithm.

In PretrainGNNs [1] framework, three pretext tasks are proposed. For node-level context prediction task, one GNN is used to generate embedding of a central node with k-hop neighborhood, and another GNN for computing context embedding for the context graph of this central node. And the pretext task is to predict whether these two embeddings belong to the same central node. For attribute masking task, some underlying chemical rules and phenomena serve as targets of supervised learning. As for multi-task property prediction task, the authors curate a diverse set of down-stream molecular property labels to construct a supervised task for pre-training.

KPGT [2] is a recently proposed pre-training framework which can be classified into masked language models category. In this framework, the molecular graph and fingerprints of a molecule are randomly masked, and the backbone encoder is trained to predict these masked components. A Line Graph Transformer model is proposed, in which original molecular graphs are transformed into line graphs, and a variant of Transformer model is used for representation learning.

S4 Training details of PTMs

The information of pre-training dataset used by our FraSICL model and other baseline models are listed in Tab. S2. Our FraSICL model uses the least number of pre-training data, but achieves the state-of-the-art predictive performance on downstream tasks, which shows its ability of making better use of the information contained in molecules.

Table S2: Training details of the PTMs

PTM	Source of the pre-training dataset	Size
MolCLR	PubChem	10M
DMP	PubChem	10M
MEMO	GEOM-Drug	300K
GROVER	ZINC15 and ChEMBL	11M
GraphLoG	ZINC15	2M
PretrainGNNs	ZINC15 (self-supervised)	2M
	ChEMBL (supervised)	456K
KPGT	ChEMBL	2M
FraSICL	PubChem	200K

S5 Pre-training Hyperparameters

Our baseline experiments are conducted on GPU Geforce RTX 3090 with CPU E5-2667 and 256GB memory. Hyperparemeters for pre-training a FraSICL model that could achieve the predictive performance reported in the baseline experiment section of the main body of our manuscript are listed as following. Chosen of backbone GNNs and the transferring settings are also presented.

- BackboneGNN-mol: Attentive FP
- BackboneGNN-frag: Attentive FP
- FinetunePTM: False
- UseWhichView: Mol
- TransformerHiddenSize: 256
- TransformerFFNSize: 256
- TransformerNumHeads: 30
- TransformerEncoderLayers:4
- EmbeddingSize: 300
- ProjectHeadSize: 300
- DropRate: 0.2
- lr: 1e-3
- L2LossGamma: 0.01
- NTXentLossTemp: 0.1
- WeightDecay: 5
- BatchSize: 200
- GNNInputSize-mol: 300
- GNNHiddenSize-mol: 300
- GNNAtomLayers-mol: 4
- GNNMolLayers-mol:2
- GNNInputSize-frag: 300
- GNNHiddenSize-frag: 300
- GNNAtomLayers-frag: 5
- GNNMolLayers-frag: 2

S6 Finetuning hyperparameters

To transfer a pre-trained FraSICL model to downstream MPP tasks, as introduced in the main body, representations of molecules extracted by the FraSICL model is fed into a downstream MLP, which serves as a prediction head. The hyperparameters are searched by grid-search with several candidates for each parameter. The candidates are listed as following.

- MLPLayers: [ ], [64], [512], [800], [128,32], [256,64], [256,64,16], [512,128,32], [256,128,64,32]
- MLPlr: 1e-2, 1e-2.5, 1e-3, 1e-3.5, 1e-4
- MLPDropRate: 0.2, 0.3, 0.4, 0.5, 0.6
- PTMlr: 1e-3, 1e-3.5, 1e-4, 1e-5
- WeightDecay: 3, 3.5, 4, 4.5, 5
- BatchSize: 100, 200, 320

S7 Codes and Scripts

The main codes of FraSICL model is publicly available at https://github.com/ZiqiaoZhang/FraSICL. Hyperparameters for reproducing the experimental results have also been reported above. Scripts and pre-training datasets will be publicly available soon after this manuscript has been accepted. The pre-trained FraSICL model checkpoint which can achieve the best performance in our experiments will also be uploaded.

References

[1] Weihua Hu, Bowen Liu, Joseph Gomes, Marinka Zitnik, Percy Liang, Vijay Pande, and Jure Leskovec. Strategies for pre-training graph neural networks. In *8th International Conference on Learning Representations, ICLR*, 2020.

[2] Han Li, Dan Zhao, and Jianyang Zeng. Kpgt: Knowledge-guided pre-training of graph transformer for molecular property prediction. *arXiv preprint arXiv:2206.03364*, 2022.

[3] Yu Rong, Yatao Bian, Tingyang Xu, Weiyang Xie, Ying Wei, Wenbing Huang, and Junzhou Huang. Self-supervised graph transformer on large-scale molecular data. In *Advances in Neural Information Processing Systems*, volume 33, pages 12559–12571, 2020.

[4] Yuyang Wang, Jianren Wang, Zhonglin Cao, and Amir Barati Farimani. Molecular contrastive learning of representations via graph neural networks. *Nature Machine Intelligence*, 4(3):279–287, 2022.

[5] Minghao Xu, Hang Wang, Bingbing Ni, Hongyu Guo, and Jian Tang. Self-supervised graph-level representation learning with local and global structure. In *Proceedings of the 38th International Conference on Machine Learning (ICML)*, volume 139, pages 11548–11558. PMLR, 2021.

[6] Jinhua Zhu, Yingce Xia, Tao Qin, Wengang Zhou, Houqiang Li, and Tie-Yan Liu. Dual-view molecule pre-training. *CoRR*, abs/2106.10234, 2021.

[7] Yanqiao Zhu, Dingshuo Chen, Yuanqi Du, Yingze Wang, Qiang Liu, and Shu Wu. Featurizations matter: A multiview contrastive learning approach to molecular pretraining. In *ICML 2022 2nd AI for Science Workshop*, 2022.
